# Pathological Abnormalities Observed on Ultrasonography among Fishermen Associated with Male Genital Schistosomiasis (MGS) along the South Lake Malawi Shoreline in Mangochi District, Malawi

**DOI:** 10.3390/tropicalmed7080169

**Published:** 2022-08-05

**Authors:** Sekeleghe A. Kayuni, Mohammad H. Al-Harbi, Peter Makaula, Boniface Injesi, Bright Mainga, Fanuel Lampiao, Lazarus Juziwelo, E. James LaCourse, J. Russell Stothard

**Affiliations:** 1 Department of Tropical Disease Biology, Liverpool School of Tropical Medicine, Liverpool L3 5QA, UK; 2 MASM Medi Clinics Limited, Medical Society of Malawi (MASM), Lilongwe P.O. Box 1254, Malawi; 3 Research for Health, Environment and Development (RHED), Mangochi P.O. Box 345, Malawi; 4 Laboratory Department, Mangochi District Hospital, Mangochi District Assembly, Mangochi P.O. Box 1854, Malawi; 5 Physiology Department, School of Life Sciences and Allied Health Professions, Kamuzu University of Health Sciences, Mahatma Gandhi Road, Blantyre 312225, Malawi; 6 National Schistosomiasis and Soil-Transmitted Helminths Control Programme, Community Health Sciences Unit, Ministry of Health, Lilongwe P.O. Box 30377, Malawi

**Keywords:** MGS, ultrasonography, prostate, epididymis, testis, seminal vesicles

## Abstract

Schistosome eggs cause granulomata and pathological abnormalities, detectable with non-invasive radiological techniques such as ultrasonography which could be useful in male genital schistosomiasis (MGS). As part of our novel MGS study among fishermen along Lake Malawi, we describe pathologies observed on ultrasonography and praziquantel (PZQ) treatment over time. Fishermen aged 18+ years were recruited, submitted urine and semen for parasitological and molecular testing, and thereafter, transabdominal pelvic and scrotal ultrasonography, assessing pathologies in the prostate, seminal vesicles, epididymis and testes. Standard PZQ treatment and follow-up invitation at 1-, 3-, 6- and 12-months’ time-points were offered. A total of 130 recruited fishermen underwent ultrasonography at baseline (median age: 32.0 years); 27 (20.9%, *n* = 129) had *S. haematobium* eggs in urine (median: 1.0 egg/10 mL), 10 (12.3%, *n* = 81) in semen (defined as MGS, median: 2.9 eggs/mL ejaculate) and 16 (28.1%, *n* = 57) had a positive seminal *Schistosoma* real-time PCR. At baseline, 9 fishermen (6.9%, *n* = 130) had abnormalities, with 2 positive MGS having prostatic and testicular nodules. Fewer abnormalities were observed on follow-up. In conclusion, pathologies detected in male genitalia by ultrasonography can describe MGS morbidity in those with positive parasitological and molecular findings. Ultrasonography advances and accessibility in endemic areas can support monitoring of pathologies’ resolution after treatment.

## 1. Introduction

Schistosomiasis is a prevalent parasitic disease in SSA where more than 90% of infected people live, causing considerable morbidity and some deaths [1,2]. Male genital schistosomiasis (MGS) is a specific-gender manifestation of urogenital schistosomiasis (UGS), associated with schistosome eggs and related pathologies in genitalia of men inhabiting or visiting endemic areas in SSA [3,4]. Despite the first recognised report described by Madden in 1911 [5], MGS remains poorly recognised and described owing to limited research over several decades.

Schistosome eggs evoke immunological responses causing granulomata formation and pathological lesions which give characteristic manifestations in the urinary tract and the liver, detectable by non-invasive radiological techniques such as ultrasonography which has become widely available in endemic areas [6]. This technique is safe, effective and can be a valuable diagnostic method in tropical diseases, such as the control of NTDs [7,8].

Ultrasonography is now portable and can be easily moved from one health facility to another, potentially supporting the semen microscopy and other available point-of-care diagnostics tests for schistosomiasis.

Transrectal ultrasonography (TRUS), computed tomography (CT) and magnetic resonance imaging (MRI) have been noted to be useful in schistosomiasis of urinary tract and liver [9]. These methods detect lesions concomitant with granulomata and calcifications, can determine pathology of various organs, as well as demonstrate calcified schistosome ova in the tissues [10,11,12,13].

Such pathologies have also been anecdotally observed to be correlated with clinical features specific to MGS and noted to resolve in earlier stages compared to irreversible, late presentation [14,15]. Furthermore, Niamey ultrasonography criteria was developed by World Health Organisation (WHO, Geneva, Switzerland) to describe the pathological lesions in renal tract and liver, associated with urogenital schistosomiasis (UGS) as well as intestinal schistosomiasis [8]. Currently there is very limited evidence in literature from few case reports of scrotal/lower abdominal studies in travellers describing MGS and impact on ultrasound pathologies of PZQ in an endemic setting, which necessitate for further studies to validate the potential use of ultrasonography in MGS and understand the evolution and resolution of the associated genital pathologies.

As part of our novel longitudinal cohort study on MGS among fishermen in south-western shoreline of Lake Malawi, known to harbour both urogenital and intestinal schistosome species [16,17], a study was conducted to describe the pathological abnormalities observed on ultrasonography over time, which could be attributed to MGS and effects of PZQ treatment(s).

## 2. Materials and Methods

### 2.1. Study Area, Population and Sampling

**Figure 1:**
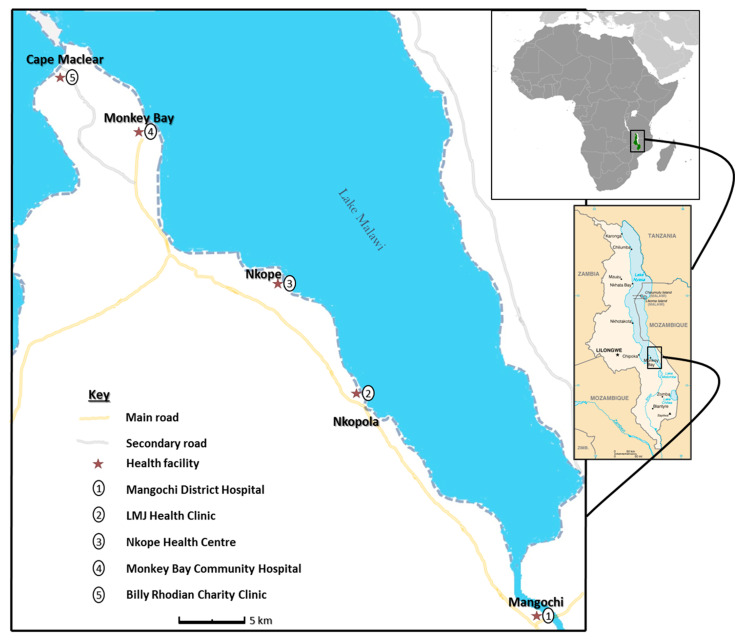
Schematic map of Study area showing health facilities along Lake Malawi. (The study map was produced by Sekeleghe Kayuni (on 4 August 2019), while the maps of Africa and Malawi were reproduced from the maps at the Central Intelligence Agency (CIA) website, public domain: https://www.cia.gov/library/publications/the-world-factbook/attachments/locator-maps/MI-locator-map.gif accessed on 4 August 2019 and https://www.cia.gov/library/publications/the-world-factbook/attachments/maps/MI-map.gif accessed on 4 August 2019).

The research study was conducted among fishermen living in fishing communities (villages) identified and selected along the southern shoreline of Lake Malawi in Mangochi district, the largest district in southern region of Malawi, from October 2017 to December 2018 (Figure 1). Fishermen aged ≥ 18 years willing to provide written informed consent were eligible to participate in the study, as described in the earlier publications [18,19].

### 2.2. Study Data Collection

**Figure 2:**
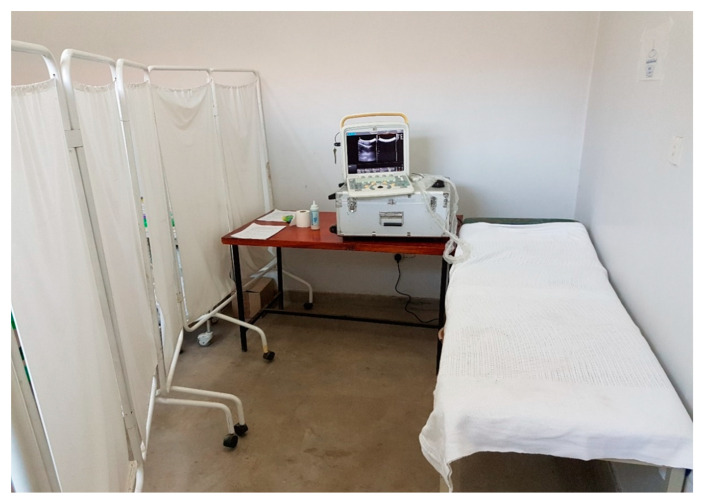
The portable Chison Q5 ultrasound scanner in an examination room.

The study methods used for the data collection included individual questionnaires, parasitological analyses of urine and semen samples collected in health facilities [18,19]. In addition, all participants were invited to undergo transabdominal pelvic and scrotal ultrasonography examination using a portable Chison Q5 ultrasound scanner with 3.5 MHz probe (Mount International United Services Ltd., Gloucester, UK) to assess pathological abnormalities in the prostate, seminal vesicles, testes and epididymis (Figure 2). The endorectal probe which is the standard of care for such examinations was considered too invasive for this study and would not be widely available outside a study context.

### 2.3. Ultrasonography Examination of Urogenital Organs

#### 2.3.1. Preparations for the Procedure

Safety precautions including use of appropriate protective wear and gloves were ensured during the ultrasonography examinations. Participants were briefed on the transabdominal and scrotal ultrasonography procedures. The scanner was set on the urology exam mode for the procedure. Participants were asked to present with a full bladder, before the procedure to increase the quality of the images. Whenever possible, room lightning was turned off to maximise screen visibility.

#### 2.3.2. Outline of the Ultrasonographic Procedure

The participant’s study number was registered in the ultrasound machine and report form prior to commencing the procedure. The participant was positioned supine on the examination couch. The scanning procedure investigated the urinary bladder, seminal vesicles and scrotum (testis, epididymis). Image quality was recorded first and then absence/presence of pathological findings were documented.

##### Urinary Bladder and Kidneys

Transverse (TS) and longitudinal (LS)sweeps through the bladder were performed to assess the shape (distension) and wall thickness, as well as the distal ureters where possible.

Schistosomiasis-related bladder pathologies included a rounded or irregular shape of the bladder, wall thickening with diffused or focal thickening of >5 mm (mild: 6–10 mm; severe: ≥11 mm), bladder wall calcifications and masses or pseudopolyps protruding in the bladder lumen; the distal ureters were considered pathological when dilated.

After performing several sweeps through the bladder, the best representative sweep was stored as a video. Bladder wall thickness was measured in mm and stored as a separate still image. In case of any pathologic findings, additional still images with relevant measurements were stored. If the bladder wall thickness was abnormal, the kidneys were scanned for evidence of hydronephrosis.

##### Prostate

The prostate was visualised during scanning of the bladder. Normal volume was set at 30 mm^3^ or less with smooth outline. Pathological findings potentially consistent with of schistosomiasis included nodules or masses larger than 1 cm, and calcifications of the prostate. After performing several sweeps through the prostate, the best representative sweep was stored under the label “prostate”. In case of any pathologic findings, additional still images with relevant measurements were stored.

##### Seminal Vesicles

Seminal vesicles were scanned in the TS plane. Normal appearances were defined as the seminal vesicles being symmetrical and measuring 15 mm or less in antero-posterior (AP) dimension with a smooth outline. Pathological findings potentially consistent with schistosomiasis were defined as enlarged and/or asymmetrical vesicles with a nodular, hyperechoic appearance.

Storage of images and clips: if the vesicles measured larger than 15 mm in AP plane, their measurement were stored as a separate still image. Following performing several sweeps through the vesicles, the best representative sweep was stored under the label “SV”. In case of any pathologic findings, additional still images with relevant measurements were stored.

##### Scrotum

Transverse sweeps of the scrotum were performed to assess both testes.

Testis abnormalities potentially suggestive of schistosomiasis were defined as nodules or calcifications; any other abnormalities of testis, epididymis and scrotum were also documented.

### 2.4. Disinfection and Patient Information after the Completion of the Procedure

At the end of the procedure the probe was cleaned with tissue paper to remove the gel, and with methylated spirit. All participants were notified of pathological findings that day by the study clinician, and further appropriate investigations and management were organised in accordance with the standard clinical practice. Thereafter, praziquantel treatment at 40 mg/kg as a single dose was offered along with an invitation to follow-up studies after 1-, 3-, 6- and 12-months.

### 2.5. Statistical Analyses

All the information collected during the study was screened and quality-controlled before entry into Microsoft Excel and SSPS computer packages. No double data entry was conducted. Screening for errors using descriptive analyses and cleaning were conducted, before commencing statistical analyses to present the results of the study. All video clips and digital images were coded for data protection and were then stored onto the device before transferring to a password-protected external hard drive for further analyses. A sample of 15% of the scan images was randomly selected and re-read by specialist radiologist for quality control, who conducted training on ultrasonography of urogenital organs.

All video clips and digital images collected from ultrasonography were stored onto the device before transferring to the external hard drive for further analyses. The data collected from the clips, images and report forms were screened to clear all errors before entry into IBM SSPS programmes in line with previous findings on specific ultrasonography [8,13,20]. Summary statistics were calculated to explore the data and thereafter correlations and significant tests were conducted to describe and interpret the results further, mainly using nonparametric tests.

### 2.6. Ethical Considerations

Ethical clearance to conduct the study was provided by the National Health Sciences Research Committee (NHSRC, approval number: 1805) of Malawi and Liverpool School of Tropical Medicine (LSTM) Research Ethics Committee (LSTM REC, approval number: 17-018), as outlined earlier. Utmost privacy and confidentiality were maintained in the study and where necessary, the information was anonymised to protect the identity of the participant.

Since this was a test-and-treat study, participants were notified of the ultrasonography results including pathological findings at the end of the procedure and where necessary, further appropriate investigations and management were organised in accordance to the standard clinical practice. Treatment with PZQ at 40 mg/kg as a single dose was offered before inviting them to the next follow-up studies at 1-month, 3-, 6- and 12-months’ time-points. Details of observed treatment were recorded when subsequent follow-ups were performed.

## 3. Results

Of the 376 fishermen recruited into the study from 39 villages located in two Traditional Authorities (T/A) of Mponda and Nankumba, only 130 participants returned to the health facility for the ultrasonography examinations at baseline of the study.

### 3.1. Demographic Information and Diagnostic Results

The median age of the 130 scanned participants was 32.0 years with a range of 19.0 to 70.0 years (Interquartile range [IQR]: 18) and their duration of stay in the fishing village ranged from 2 months to 70 years (median: 22.0; IQR: 24.5; Table 1). The median weight of the participants was 59.0 kg (IQR: 9.0, range: 43.0–75.4 kg).

All participants except one submitted urine and 81 submitted semen (62.3%). After urine filtration, 27 participants (20.9%) had *S. haematobium* eggs in urine (UGS), their mean egg count was 19.1 eggs per 10 mL and ranging from 0.1 to 186.0 eggs (median: 1.0, IQR: 5.8). Six participants (4.9%) had a positive POC-CCA test, suggestive of possible intestinal *S. mansoni* infection.

For the 81 participants who submitted semen, 10 (12.3%) had *S. haematobium* eggs in semen (MGS), mean egg count was 3.9 per ml of ejaculate (median: 2.9 eggs), ranging from 0.4 to 9.3 eggs and volume of semen ranged from 0.1 to 4.5 mL (mean: 1.6 mL). The real-time PCR conducted on 57 semen samples revealed that 16 participants (28.1%) were positive. Four participants were positive on both semen microscopy and real-time PCR.

### 3.2. Baseline Results of the Ultrasonography Exanimations

Of the participants who had ultrasonography, 9 (6.9%) participants had abnormalities in genital organs. Specifically, abnormalities were noted in prostate, seminal vesicles and/or scrotum (testis and epididymis) (Table 2). One participant who had abnormalities in urinary bladder wall with severe polypoid thickness was detected to have bilateral hydronephrosis (Table 3).

Eighteen of all the scanned participants had UGS only, confirmed by *S. haematobium* eggs observed in urine, 15 had MGS only (six had semen eggs only while 9 were positive for real-time PCR) and only two participants were positive for all three diagnostic tests. Only 4 participants with sonographic abnormalities had MGS confirmed by semen microscopy and real-time PCR, two participants had schistosome eggs in urine and semen and positive real-time PCR.

Of the four participants with abnormalities in at least 2 GU organs as shown in Table 3 below, the abnormalities of 2 with confirmed MGS could be classical description of its morbidity (participants 2 and 3):

#### 3.2.1. Urinary Bladder and Kidneys

Two participants with abnormal ultrasound findings had irregular outline of their urinary bladder with severe wall thickness of at least 11 mm, one had bilateral hydronephrosis (participant 1, Table 3) while the other (participant 2, Table 3) had severe focal bladder thickness.

#### 3.2.2. Prostate

**Figure 3:**
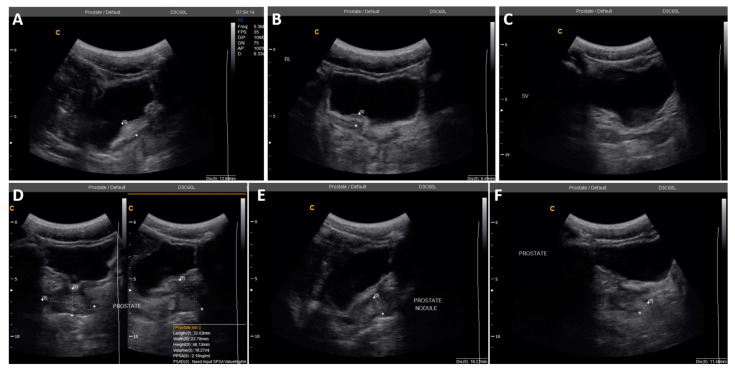
Ultrasonographic images of an MGS positive study participant with abnormalities in the bladder and prostate at Baseline. (**A**,**B**). Irregular urinary bladder wall and severe focal thickness, measuring up to 13.6 mm. (**C**). Normal symmetrical seminal vesicles. (**D**). Prostate with abnormal irregular outline, but normal volume of 18.3 mL. (**E**,**F**). Hyperechoic nodule in the prostate, measuring 11.4 mm by 16.2 mm.

**Figure 4:**
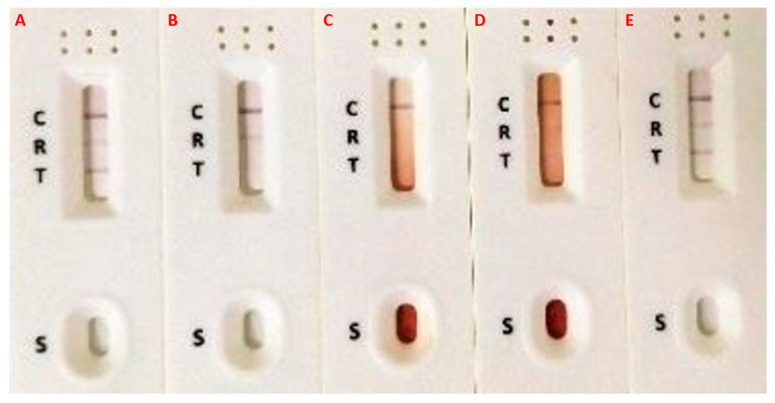
Images of the POC-PSA test conducted on participants with Prostate abnormalities. (**A**,**E**). Strong positive POC-PSA with no urine or semen eggs and no abnormalities. (**B**). Negative POC-PSA with semen real-time PCR (Ct-value: 25.4), irregular prostate outline and hyperechoic nodule. (**C**). Negative POC-PSA with no urine eggs, but had grossly irregular, enlarged prostate (61.3 mL), abnormal epididymis and bilateral hydrocele. (**D**). Negative POC-PSA with no urine or semen eggs, negative real-time PCR but had irregular, enlarged prostate (39.1 mL).

Three participants (2.3%) had abnormal prostate appearances, two aged 51 and 69 years old, had enlarged prostates with volumes of 39.1 mL and 61.3 mL. The other participant, aged 22 years with confirmed MGS through positive semen real-time PCR, had irregular prostate outline and hyperechoic nodule (Figure 3), attributable to description of MGS morbidity. A point-of-care prostate specific antigen (POC-PSA) conducted on these participants was negative, excluding possibility of prostatic infection, hyperplasia or tumour (Figure 4).

#### 3.2.3. Seminal Vesicles

Only one participant had asymmetrical, hyperechoic vesicles, suggestive of MGS related pathology, aged 24 years with *S. haematobium* eggs in urine and semen, as well as positive real-time PCR. The measurements for the scanned participants’ right vesicle were from 4.8 mm to 18.5 mm and the left vesicle from 3.7 mm to 17.5 mm.

#### 3.2.4. Scrotum

**Figure 5:**
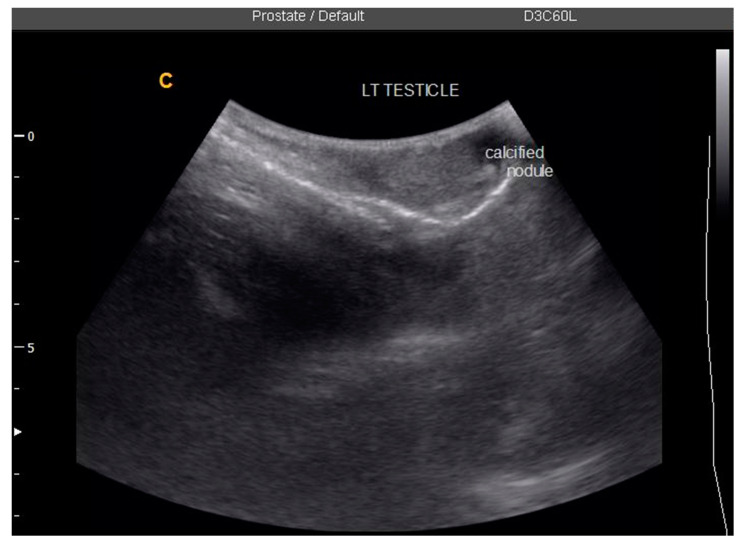
Ultrasonographic image of study participant with left testicular nodule at baseline.

One participant (0.8%) had a left testicular nodule, with *S. haematobium* eggs detected in urine and semen (Figure 5). No testicular abnormalities were detected in the other MGS participants. Only one participant, aged 69, was observed to have an abnormal enlarged right epididymis (participant 4, Table 3), which could be attributable to MGS morbidity.

In context with all the abnormalities described above, there was no correlation between age, duration of stay, diagnostic tests’ results and abnormalities.

### 3.3. Follow-Up Ultrasonography Examinations

At the end of the ultrasonography examinations at baseline, PZQ treatment was provided to the participants on exit of the study after submitting semen sample for resolution of the abnormalities. The participants were invited to follow-up ultrasonography examinations at 1-, 3-, 6- and 12 months’ time-points.

#### 3.3.1. One-Month Follow-Up

Only 29 participants were scanned out of the 60 participants who returned at 1-month follow-up, with four participants scanned for the first time and no abnormalities observed. Only one of two participants with classical description of MGS morbidity was scanned at this time-point and had bilateral hydroceles despite PZQ treatment and negative tests (Table 4).

#### 3.3.2. Three-Months Follow-Up

Sixty-four participants were followed up at 3-months’ time-point of which 32 had ultrasonography examinations, and 4 were scanned for the first time. On diagnostic examinations, 5 had *S. haematobium* eggs in urine (17.2%), 4 in semen (13.8%), 4 had trace POC-CCA test while 5 had positive semen real-time PCR. 

The MGS participant with prostate nodule at baseline had no abnormality detected while the testicular nodule participant was lost-to-follow-up. Three other participants (28.1%) had hydroceles at this time-point.

#### 3.3.3. Six-Months Follow-Up

Sixty-three participants were followed up at 6-months’ time-point of which 38 had ultrasonography examinations, and 4 were scanned for the first time. On diagnostic examinations, 2 had *S. haematobium* eggs in urine (5.3%), 1 in semen (2.6%), 4 (10.5%) positive and 2 (5.3%) trace POC-CCA test results, while 3 had positive semen real-time PCR (7.6%). 

The MGS participant with prostatic nodule at MGS was lost to follow-up, while a 33-year-old participant with negative diagnostic tests had enlarged seminal vesicles and other three had hydroceles.

#### 3.3.4. Twelve-Months Follow-Up

Forty-five participants were reviewed at 12-months’ time-point of which 17 had ultrasonography examinations, and 4 were scanned for the first time. One participant had abnormal bladder wall thickness and left kidney mass. On diagnostic examinations, the participants were negative for POC-CCA, urine filtration and semen microscopy, with 2 participants being positive on semen real-time PCR (11.8%).

Table A1 (in Appendix A) illustrates the progress of abnormalities detected at baseline, over the course of the study and Table A2 shows those with no abnormality at baseline but detected during the follow-up. In total, 146 participants were scanned in the study and abnormalities were noted in 16 participants at various time points (Table A3).

## 4. Discussion

To our knowledge, this is the first prospective ultrasonographic study of MGS in Malawi and South-eastern Africa on the fishermen cohort to determine its morbidity, through observations of pathological abnormalities in genital organs, as well as look at changes through time in men after standard dose-regimen of PZQ treatment. Our study observed classical abnormalities which could describe the morbidity of MGS, namely prostatic and testicular nodules in two confirmed participants on parasitological and molecular testing.

### 4.1. Genital Consequences of Schistosomiasis on Ultrasonography

Genital manifestations such as MGS are among the complications of schistosomiasis which remains unknown among local inhabitants frequently exposed to infective cercariae harbouring their freshwater bodies as well as health professionals working in the areas which result in undiagnosed, under- or mistreating and underreporting of the disease, contributing further to morbidity among affected men. In some instances, people suffer from social prejudice and discrimination arising from the consequences of the genital complications, such as infertility, abnormal organ swelling, reduced sexual prowess, coital pain, genital bleeding among other with women severely and disproportionately [21,22].

In order to improve awareness and knowledge of MGS, other diagnostic methods can be added to help in detection of the disease. Radiological techniques have been observed to improve diagnosis of schistosomiasis of liver and urological tract at various stages, mostly with chronic complications. Ultrasonography is considered as an acceptable, safe and less-invasive tool in diagnosis, management and monitoring control of NTDs, including schistosomiasis. Recent advances in this technology have resulted in development of portable, high quality scanning devices which can be easily mobile to limited-resource endemic areas and utilised in the available infrastructure, in detecting the genital pathological abnormalities affecting rural population among other conditions, as demonstrated in our study.

### 4.2. Pathological Abnormalities Associated with MGS in Malawian Fishermen

Our MGS cohort study among local fishermen along the south shoreline of Lake Malawi observed a 17.1% baseline prevalence of UGS, 10.4% for MGS using semen microscopy and 26.6% by semen real-time PCR. Among those 130 participants who were scanned at baseline, the prevalence of MGS was 12.3% using semen microscopy and 28.1% by semen real-time PCR.

Previous studies have described abnormalities observed in genital organs such as prostate, seminal vesicles, ejaculatory ducts, vas deferens, epididymis, tests, scrotal sac among other structures, which include organ enlargement, shrinkage, dilatations, thickening, echogenic lesions, calcification, hydroceles among others, which can mimic other diseases. In schistosomiasis-endemic areas, detection of such pathological abnormalities together with classical genital symptoms such as genital, coital or ejaculatory pain, haemospermia, abnormal ejaculates, reduced libido or suspected infertility, could suggests a diagnosis of MGS [19]. Since similar clinical presentation and findings can be associated with other diseases prevalent in these endemic areas, affecting the genital organs such as sexually transmitted infections (STIs), tuberculosis (TB), malignant hypertension or cancer, proper clinical assessment and extensive diagnostic examinations are very important to be conducted to ensure appropriate diagnosis, treatment, care and management of affected men.

The findings of this study showed that ten participants had pathological abnormalities in their GU organs at baseline, of which nine participants (6.9%) having them in prostate, seminal vesicles and scrotum and two had classical abnormalities, descriptive of MGS morbidity. These abnormalities suggest consequences of previous or current schistosomal infection acquired from their frequent exposure during fishing and other routine activities in the lake, which is known to harbour schistosomes. Seminal vesicles were observed to have abnormalities in the study, consistent with evidence from previous studies and literature which describes that seminal vesicles are among the frequent affected genital organs with schistosomiasis [15,23,24].

Further results show that urinary bladder had abnormal wall thickening, in some cases severe polypoid structures and associated bilateral hydronephrosis which required referral for further medical management at district hospital, unfortunately, participant was lost to follow-up. Such lesions have been widely described in the literature originating from schistosome worms which have matured as male and female worms in hepatic venules, pair up and migrate to the vesical plexus where they reside around urinary bladder. These worms continually deposit massive number of schistosome eggs, which get trapped in the bladder wall and cause granulomatous reactions, fibrosis, calcifications and architecture destruction, thereby compromising bladder functioning [25,26]. As the infection progresses and bladder malfunctions, urine backflow compromises the ureters (hydroureter) which later affects the kidneys, resulting in hydronephrosis. Early diagnosis and management are critical in preventing such chronic and fatal consequences of schistosomiasis.

Interestingly, schistosome worms have been thought to reside in venous plexus around the genital organs such as prostate, seminal vesicle and testes, with eggs being trapped in the tissues due to its tough architecture in comparison to the urinary bladder. This can result in echogenic lesions, calcifications, organ enlargement, atrophy, hydroceles among the pathological abnormalities in these genital organs which can be detected on ultrasonography [11,14,27]. These abnormalities were observed in this study in the prostate, epididymis and testis, with some participants being positive on the urine and semen diagnostic tests. Prostate abnormalities were observed in three participants, of which two had grossly enlarged prostates and one had hyperechoic prostatic nodule and positive semen real-time PCR. These can be attributed to MGS after exclusion of other possible diseases such as STIs, TB, benign prostatic hyperplasia or prostate malignancy. In addition, the lack of statistically significant differences among those with abnormalities in accordance to age, duration of stay and diagnostic tests’ results demonstrate that the lesions can present at any age since they could have developed from a young age, as reported previously [28,29].

### 4.3. Pathological Abnormalities after Treatment

Monitoring of disease morbidity especially MGS is critical in controlling the disease and prevention of severe irreversible pathological abnormalities which later could contribute to mortality. As a mainstay treatment, PZQ has shown to be effective in treating both forms of schistosomiasis, registering cure rates of over 90% in most endemic areas [30,31]. Currently, it is utilised by most national control programmes in endemic areas as one of the key control interventions through the MDA campaigns, which commonly targeted school-aged children. PZQ has also been used in treating MGS, clearing the schistosome eggs in semen and resolving some pathological abnormalities, while adjusting the standard dose of treatment in some cases to ensure complete cure [15].

From the study follow-up after PZQ treatment to the participants, pathological abnormalities were not detectable in most participants on follow-ups. This could illustrate the knowledge that early mild abnormalities will be resolved by standard PZQ treatment as it kills those adult-laying worms, hence reducing further damage to the organs. Other chronic, long-standing abnormalities such as hydroceles detected on follow-up, required further assessments, medical and surgical interventions to resolve these, as it was done in the study where such participants were referred to the bigger district hospital.

Repeated exposure to the infested lake water can contribute to newer abnormalities developing after PZQ treatment. Moreover, longer duration in resolution of abnormalities support the need for repeated PZQ treatment to completely resolve the severe abnormalities which are reversible, while providing additional control interventions such as adequate awareness and health education, provision of adequate, portable, safe and clean water, encouraging construction and utilisation of household and community sanitation facilities, as well environmental control to reduce intermediate snail host population.

### 4.4. Study Limitations and Ultrasonographic Diagnostic Challenges in MGS

The low number of participants undergoing the ultrasonography examinations and inadequate volumes of semen samples to run real-time PCR limit the tests results’ comparisons and generalisation of the study results to male population in endemic region. This could be explained by lack of experience with the method as most participants reported this was their first time to undergo such examinations. Negative perceptions with regards to new techniques in rural communities and the longer time spent during the examination especially among those presenting with inadequate bladder filling could have deterred more study participants from taking part. Moreover, some participants could be reluctant to take part at health centres, due to poor health-seeking behaviour. More sensitisation and discussions which were done can help to address such and other concerns, thereby stimulate more participants to such important studies. 

The low sensitivity of transabdominal ultrasonography compared to TRUS, CT or MRI could result in missing some lesions in the genital organs, resulting in poorly described burden of genital diseases such as MGS. However, cost implications associated with these sensitive techniques, their unavailability and inaccessibility as well as low acceptability among local participants could further jeopardise the implementations of such examinations in rural endemic areas.

In addition, advanced radiological expertise and extensive training on genital ultrasonography are required in order to detect pathological abnormalities arising from MGS, which could be easily mistaken for those from other prevalent genital diseases such as STIs, TB or cancer. Moreover, further ultrasonography studies with larger cohort as well as inclusion of more advanced portable devices such as TRUS are necessary in endemic areas to describe more on morbidity of MGS.

## 5. Conclusions

In conclusion, pathological abnormalities can be detected using portable transabdominal and scrotal ultrasonography, as demonstrated in our study, which together with positive parasitological and molecular MGS tests could describe its morbidity. Owing to advances in portable ultrasonography and their accessibility in endemic areas especially SSA, together with symptomatology description and parasitological findings, this diagnostic technique can also aid in monitoring in MGS treatment and resolution of its related pathologies.

## Figures and Tables

**Table 1:** Demographical information, diagnostic analyses on urine and semen results of the 130 study participants.

Variable	*n*	Median	Range	Interquartile Range (IQR)	Number of Positive Cases	Prevalence (%)
Age	130	32.0	19.0–70.0	18.0	-	-
Duration of stay in village (years)	120	22.0	0.2–70.0	24.5	-	-
Weight (kgs)	115	59.0	43.0–75.4	9.0	-	-
Eggs in urine (filtration, 10 mL)	129	1.0	0.1–186.0	5.8	27	20.9
POC-CCA	129	-	-	-	6	4.9
Eggs in semen (mL)	81	2.9	0.4–9.3	4.6	10	12.3
Seminal real-time PCR (Ct-value)	57	26.4	18.9–36.6	10.5	16	28.1%

**Table 2:** Proportion of abnormal findings in particular organs at baseline.

Organ	Total Scans	Number of Abnormal Scans	Percentage (%)
Urinary Bladder *	106	2	1.9%
Prostate	126	3	2.4%
Seminal vesicles	117	1	0.9%
Testis ^#^	129	1	0.8%
Epididymis ^ǂ^	129	1	0.8%
Scrotum ^**α**^	129	6	4.7%

* Bilateral hydronephrosis in 1 participant, **^#^** left testis; ^ǂ^ right epididymis; **^α^** hydroceles were observed in scrotums of six participants.

**Table 3:** Abnormalities observed in participants with at least 2 GU organs affected at baseline.

Participant	Age (Years)	Eggs in Urine (per 10 mL)	Eggs in Semen (per mL)	Real-Time PCR (Ct-Value)	Abnormalities Observed
1	19	0	0	N/D ^#^	Irregular bladder wall and severe polypoid thickness, with bilateral hydronephrosis
2	22	0	0	25.4	Irregular bladder wall with severe focal thickness, irregular prostate with hyperechoic nodule (Figure 3)
3	49	1	6	N/D ^#^	Left testicular nodule and mild bilateral hydroceles
4	69	0	N/A ^ǂ^	N/A ^ǂ^	Severely enlarged prostate (volume = 61.3 mL) and right epididymis, with bilateral hydrocele

**^#^** Test not done, inadequate sample; ^ǂ^ sample not submitted, test not done.

**Table 4:** Observations on ultrasonography of one participant at baseline and one-month follow-up.

Age (Years)	Baseline		1-Month Follow-Up	
	Test Results	Abnormalities Observed	Test Results	Abnormalities Observed
49	Eggs in urine, semen; no real-time PCR done	Left testicular nodule, mild bilateral hydroceles	No eggs in urine or semen; negative PCR	Bilateral hydroceles

## Data Availability

The data for this study has been presented within this article and any further information regarding this study can be reasonably requested from the corresponding author.

## Appendix A

**Table A1:** Ten participants with abnormalities in Pelvic organs (9 in genital organs) on ultrasonography at baseline and progression over the time points in the study.

Age (Years)	Baseline	1-Month	3-Months	6-Months	12-Months
19	Bladder thickening, severe hydronephrosis	Lost to follow-up			
22	Bladder thickening, prostate nodule	Did not show up	No abnormality	Inadequate bladder filling, not reported for repeat scan	Lost to follow-up
24	Enlarged asymmetrical seminal vesicles	Inadequate bladder filling, not reported for repeat scan	Lost to follow-up		
29	Left hydrocele	Lost to follow-up			
30	Left hydrocele	Did not show up	Did not show up	Did not show up	No abnormality
49	Bilateral hydroceles	Bilateral hydroceles	Bilateral hydroceles	Bilateral hydroceles	Lost to follow-up
49	Testicular nodule, mild bilateral hydroceles	Bilateral hydroceles	Lost to follow-up		
51	Enlarged prostate	Did not show up	Did not show up	No abnormality	Lost to follow-up
54	Bilateral hydroceles	Lost to follow-up			
69	Enlarged prostate, bilateral hydroceles	Lost to follow-up			

**Table A2:** Six participants with abnormalities in GU organs on ultrasonography during follow-up time-points but none at baseline in the study.

Age (Years)	Baseline	1-Month	3-Months	6-Months	12-Months
25	No abnormality	No abnormality	*Did not show up*	Left hydrocele	Lost to follow-up
33	No abnormality	*Did not show up*	Left hydrocele	*Did not show up*	No abnormality
33	No abnormality	*Did not show up*	*Did not show up*	Enlarged, asymmetrical seminal vesicles	Lost to follow-up
39	No abnormality	*Did not show up*	*Did not show up*	Left hydrocele	Lost to follow-up
44	No abnormality	Left hydrocele	Left hydrocele	No abnormality	Lost to follow-up
53	No abnormality	*Did not show up*	*Did not show up*	*Did not show up*	Bladder thickening, left kidney mass

**Table A3:** Proportion of participants in the study with abnormalities in GU organs on ultrasonography in accordance with their MGS status at all time-points.

		MGS									
		Baseline (*n* = 130)		1-Month (*n* = 29)		3-Months (*n* = 32)		6-Months (*n* = 38)		12-Months (*n* = 17)	
		Positive	Negative	Positive	Negative	Positive	Negative	Positive	Negative	Positive	Negative
**USS**	**Positive**	4	5	2	1	1	2	0	4	0	0
	**Negative**	14	107	1	25	3	26	1	33	2	15
